# Clinical and echocardiographic features of aorto-atrial fistulas

**DOI:** 10.1186/1476-7120-3-1

**Published:** 2005-01-17

**Authors:** Karthik Ananthasubramaniam

**Affiliations:** 1 Henry Ford Heart and Vascular Institute, Detroit MI 48202, USA

## Abstract

Aorto-atrial fistulas (AAF) are rare but important pathophysiologic conditions of the aorta and have varied presentations such as acute pulmonary edema, chronic heart failure and incidental detection of the fistula. A variety of mechanisms such as aortic dissection, endocarditis with pseudoaneurysm formation, post surgical scenarios or trauma may precipitate the fistula formation. With increasing survival of patients, particularly following complex aortic reconstructive surgeries and redo valve surgeries, recognition of this complication, its clinical features and echocardiographic diagnosis is important. Since physical exam in this condition may be misleading, echocardiography serves as the cornerstone for diagnosis. The case below illustrates aorto-left atrial fistula formation following redo aortic valve surgery with slowly progressive symptoms of heart failure. A brief review of the existing literature of this entity is presented including emphasis on echocardiographic diagnosis and treatment.

## Case

A 66 year old male with history of rheumatic heart disease and aortic valve replacement (AVR) (twice for severe native and subsequent prosthetic valve regurgitation) presented with progressive worsening fatigue, exertional dyspnea and paroxysmal nocturnal dyspnea. His other medical problems included poorly controlled hypertension and hyperlipidemia. Following his second AVR 4 years prior to this presentation, a routine follow-up 2-dimensional echocardiogram (TTE) had shown preserved left ventricular and prosthetic valve function and a aorto-atrial fistula with color flow between the aorta-and the left atrium. Notably, only a soft and short ejection murmur across the mechanical prosthesis was appreciated and no continuous murmurs were heard. This was felt to be a possible postoperative complication currently not of clinical significance given his asymptomatic status and he was treated medically and did well for the last 3–4 years.

**Figure 1:**
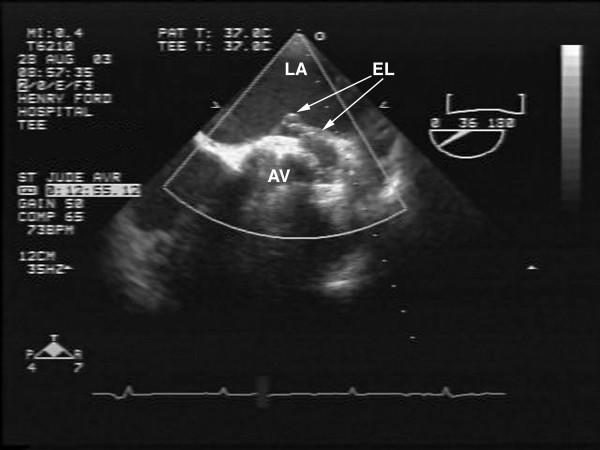
Off axis 2-D short axis TEE view demonstrates the left atrium (LA) the prosthetic aortic valve (AV). An echolucent area (EL) around the aortic valve protruding into LA is seen with focal outpouching into the LA. This represents weakening of the wall of the aorta near the posterior aspect of the LA

**Figure 2:**
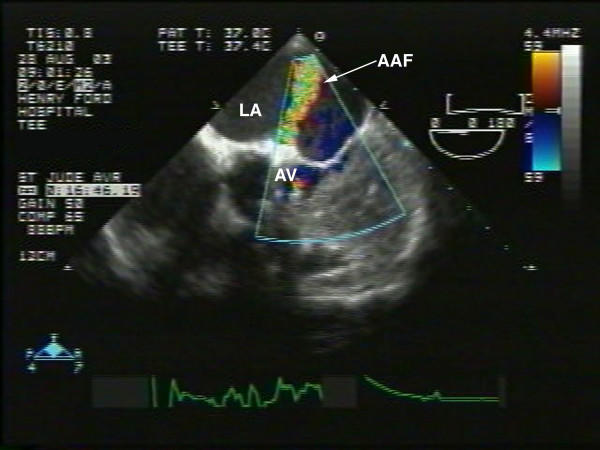
Off axis 2-D short axis TEE view with the probe advanced further into mid-esophagus: demonstrates turbulent color flow entering the LA from the EL region of the aortic valve illustrated in Fig 1

**Figure 3:**
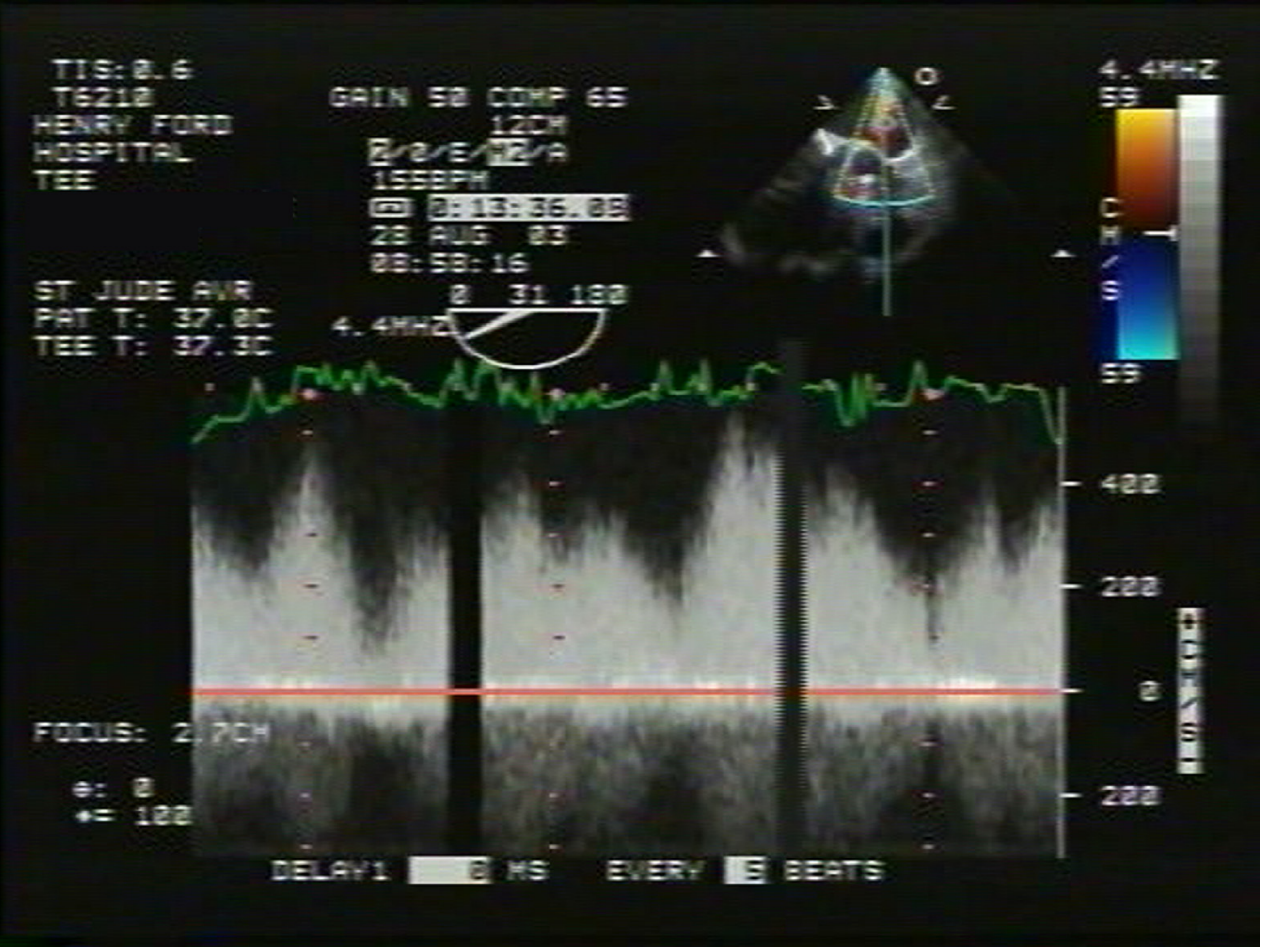
Continuous wave Doppler signal across turbulent jet showing high velocity throughout cardiac cycle but predominantly in systole consistent with aorto-left atrial fistula

Physical examination during this visit revealed an afebrile patient with a blood pressure of 138/84 mm Hg, regular pulse of 84/minute. An ejection systolic murmur (2/6 in intensity) was heard all over the precordium likely from the flow across his prosthesis. No continuous murmurs were heard. No evidence for clinical heart failure, anemia, jaundice or infection was noted. Laboratory tests revealed no leukocytosis and blood cultures were negative. Given prior echo documentation of fistula and new symptomatology suggestive of heart failure, a transesophageal echocardiography (TEE) was requested for more detailed assessment of prosthesis and AAF. TEE revealed normal left ventricular function, normal aortic prosthesis function with trivial aortic regurgitation. An echolucent area above the mechanical prosthesis, close to the left atrium near the orifice of the left coronary artery was noted. There appeared to be expansion of a portion of this lucency into the left atrium during systole suggesting communication with the aorta (Fig 1) with turbulent color flow from the aorta into the left atrium (suggestive of AAF) throughout the cardiac cycle but mainly in systole as shown by color and continuous wave doppler (Figs 2 and 3). Compared to the prior 2-D echo there appeared to be mild left atrial dilation, mild left ventricular hypertrophy and significantly more prominent fistula flow suggesting either progressive shunting and enlargement of the fistula over time or underestimation by the prior 2-D echo. Although the echocardiographic findings mimicked changes which could also be related to endocarditis (abscess around prosthesis with pseudoaneurysm formation), the absence of any obvious vegetations or prosthetic malfunction combined with lack of clinical and laboratory evidence of endocarditis favoured a more slowly progressive postoperative complication rather than an infectious process. Based on his heart failure symptoms and progressive increase in AAF size and flow, surgical correction was recommended. He underwent uncomplicated surgical repair of the AAF which was found during surgery to be inferior to the left coronary ostium. No evidence of abscess or infection was found and the prosthesis appeared intact and healthy. The echo lucent area represented a postoperative weakening of the aortic wall adjacent to the left atrium, predisposing to the fistula formation and was also repaired. Intra-operative TEE showed no residual fistula by color flow at the site of repair.

## Discussion

In a large collection of about 4000 cases of thoracic aortic aneurysms, Boyd first reported AAF as an incidental finding on autopsy back in 1924 [1]. Most of common etiologies of AAF are related to its occurrence as a result of bacterial endocarditis, paravalvular abscess, ruptured sinus of Valsalva, aortic dissection and possibly of congenital etiology [2-6].

Clinical presentation of AAF could vary from an acute presentation with acute chest pain syndrome due to rupture in the setting of dissection [5] or a refractory heart failure picture in the setting of endocarditis [7] and aortic dissection [8]. In a previous report we have highlighted the fulminant course of prosthetic valve endocarditis due to Proteus mirabilis leading to aorto-right atrial fistula from rupture of a pseudoaneurysm secondary to prosthetic valve endocarditis [9]. Isolated case reports of AAF as an immediate postoperative complication has also been reported [10].

### Role of Echocardiography

TTE and TEE form an integral part of assessment of patients presenting with chest pain and heart failure symptoms particularly if audible murmurs or valvular pathologies are suspected. TTE is the intial test of choice in routine prosthetic aortic valve assessment and gradients estimation. Nevertheless TEE is superior to TTE in real time assessment of prosthetic valve function and morphology and for better delineation of intracardiac pathology such as complications of endocarditis namely root abscess and fistulas [7,11,12]. TEE has better signal to noise ratio and proximity of transducer to the heart leading to higher quality images with lesser attenuation. Furthermore since aorto-left atrial fistulas usually occur from the posterior aspect of the aorta, this area is better delineated with TEE than TTE. Turbulent flow of AAF can be mistaken on TTE particularly if near the prosthetic valve for prosthetic malfunction in the setting of endocarditis or heart failure. Inter-chamber communications and the fistulous tracts that are particularly small are best tracked by multiplane TEE. The exact origin, chamber communications and even the size of the fistulous opening can be well assessed by TEE. Furthermore, coexistent complications with AAF in the setting of aortic endocarditis such as presence of annular abscess, extension to the upper interventricular septum or the subaortic area and pseudoaneurysm formation are best seen by TEE. Nevertheless cases of underestimation of cardiac involvement also been reported with TEE [9]. This reveals the limitations of viewing a three dimensional structure such as the heart in a two dimensional fashion, a void which may be filled by 3-dimensional (3-D) echocardiography.

This case highlights the importance of intraoperative TEE in guiding valvular surgery identifying potential intraoperative cardiac complications, which can be corrected in the same setting. Since repeat sternotomy and cardiac surgery by itself carries a higher risk of perioperative complications, intraoperative pre and post pump TEE play an integral role in guiding the surgeons as to any new complications which may have risen during surgery. This is more so in valve surgeries or aortic reconstruction surgery where a real time 2-D TEE with color assessment pre and post pump provides important information regarding success of surgical intervention and new complications.

Since clinical diagnosis of AAF is difficult, definitive diagnosis is by a thorough echocardiographic evaluation (TTE and TEE). Apart from antibiotic treatment for endocarditis, definitive treatment revolves around surgical correction. Since many of these patients may have had some form of surgical intervention in the past [5], reoperation is challenging. Mortality is high in patients who are continued on medical therapy particularly in the setting of aortic dissection [5,7]. Surgical intervention consists of repairing the affected aortic segment, replacing prosthesis if the valve is destroyed, annular debridement in the setting of abscess and suture of the fistula.

## Conclusion

Aorto atrial fistulas are rare but important complications of many disease processes of the aorta and aortic valve. Classical clinical signs of continuous murmurs may not be present and echocardiography forms the cornerstone of diagnosis. AAF should be suspected in patients with poorly controlled heart failure and prior aortic surgery. Prompt surgical repair is usually helpful in relieving symptoms and decreasing mortality.

## Competing interests

The author declares that he has no competing interests in preparation of this mansucript and has fully contributed to preparation of this manuscript.
